# Exoenzyme T Plays a Pivotal Role in the IFN-γ Production after *Pseudomonas* Challenge in IL-12 Primed Natural Killer Cells

**DOI:** 10.3389/fimmu.2017.01283

**Published:** 2017-10-10

**Authors:** Mickael Vourc’h, Antoine Roquilly, Alexis Broquet, Gaelle David, Philippe Hulin, Cedric Jacqueline, Jocelyne Caillon, Christelle Retiere, Karim Asehnoune

**Affiliations:** ^1^Laboratoire UPRES EA3826 «Thérapeutiques cliniques et expérimentales des infections», IRS2 - Nantes Biotech, Université de Nantes, Nantes, France; ^2^Intensive Care Unit, Anesthesia and Critical Care Department, Hôtel Dieu, University Hospital of Nantes, Nantes, France; ^3^Etablissement Français du Sang, Nantes, France; ^4^CRCINA, INSERM U1232, CNRS, Université d’Angers, Université de Nantes, Nantes, France; ^5^MicroPICell, Cell and Tissue Imaging Core, UMS Inserm 016/CNRS 3356/FED 4203, Villejuif, France

**Keywords:** natural killer cells, *Pseudomonas aeruginosa*, type III secretion system, innate lymphoid cells, IL-12, interferon-gamma

## Abstract

*Pseudomonas aeruginosa* (PA) expresses the type III secretion system (T3SS) and effector exoenzymes that interfere with intracellular pathways. Natural killer (NK) cells play a key role in antibacterial immunity and their activation is highly dependent on IL-12 produced by myeloid cells. We studied PA and NK cell interactions and the role of IL-12 using human peripheral blood mononuclear cells, sorted human NK cells, and a human NK cell line (NK92). We used a wild-type (WT) strain of PA (PAO1) or isogenic PA-deleted strains to delineate the role of T3SS and exoenzymes. Our hypotheses were tested *in vivo* in a PA-pneumonia mouse model. Human NK cells or NK92 cell line produced low levels of IFN-γ in response to PA without IL-12 stimulation, whereas PA significantly increased IFN-γ after IL-12 priming. The modulation of IFN-γ production by PA required bacteria-to-cell contact. Among T3SS effectors, exoenzyme T (ExoT) upregulates IFN-γ production and control ERK activation. *In vivo*, ExoT also increases IFN-γ levels and the percentage of IFN-γ^+^ NK cells in lungs during PA pneumonia, confirming *in vitro* data. In conclusion, our results suggest that T3SS could modulate the production of IFN-γ by NK cells after PA infection through ERK activation.

## Introduction

*Pseudomonas aeruginosa* (PA) is an opportunistic pathogen that causes lung infections in cystic fibrosis (CF) (1) as well as in intensive care unit (ICU) patients (2). In CF patients, PA infection appears after a few years and systematically becomes chronic, inducing severe pulmonary damage. In ICU patients, PA-related ventilator-associated pneumonia reduces survival and worsens outcome. The high level of PA recurrence is related to its high virulence and hypermutable genome (3), while the ability to subvert immunity may explain chronic infection.

*Pseudomonas aeruginosa* alters innate lymphoid cells, including natural killer (NK) cells, which play a key role in immunity against PA (4). NK cells give rise to cytokine or cytotoxic response but cytokine production prevails after bacterial infection (5). NK cells are a major source of IFN-γ, which participates in antimicrobial immunity and stimulates monocyte differentiation (6). Conversely, PA can divert cytokine response and use IFN-γ to enhance its virulence factors (7).

In order to explain how PA infection can give rise to proinflammatory response, we explored how PA can trigger IFN-γ release and especially the role of the type III secretion system (T3SS) and its effector (Exoenzyme T, S, and Y). It has been suggested that toll-like receptors (TLRs), natural cytotoxic receptors (NCRs), and killer-cell immunoglobulin-like receptors (KIRs) on NK cells can sense bacteria and trigger cytokine response (8). Alongside NK-specific pathogen recognition, antigen-presenting cells like DCs are critically involved in NK cell activation through IL-12, IL-15, IL-18, or IL-21 release (9, 10).

We sought to precisely describe the underlying mechanism of IFN-γ response in NK cells during PA infection by specifically analyzing virulence factors and pathway activation in an *in vitro* infection model. Since IL-12 is required to observe the production of IFN-γ during PA infection, we examined *in vitro* the effects of PA on the production of IFN-γ by IL-12-treated NK cells. Last, we validated our data *in vivo* in a mouse PA pneumonia model.

## Materials and Methods

### Bacterial Strains

PA01 is a clinical strain of PA (no. 15692) (11) whose genome has been fully sequenced. It expresses most of the documented virulence factors, including the T3SS also known as the “needle complex” and its effectors: Exoenzymes (Exo) S, T, and Y released in targeted cells through T3SS. Three isogenic deleted strains were used: PA-ΔS (ExoS deletion), PA-ΔT (ExoT deletion), and PA-ΔT3SS (deletion of the needle complex). PA-ΔS and ΔT were a gift from Dr. Andrew Y. Koh Laboratory at the University of Texas Southwestern Medical Center in Dallas, TX, USA. PA expressing the Green Fluorescent Protein (PA-GFP) was a gift from Dr. Wu at the University of North Dakota. PA-ΔT3SS (also called ΔPscC) carries a truncated PscC gene leading to a non-functional protein. PscC is a secretin-like constitutive protein of the outer membrane forming a channel enabling needle growth. Without the functional pscC protein, the needle in the T3SS cannot protrude to the cell surface and, as a result, the bacteria cannot inject Exo in the host cell cytoplasm (12). This strain came from Dr. Donald Moir at microbiotix^INC^ in Worcester, MA, USA. The PCR study confirmed the phenotype of each deleted strain (see Figure S1 in Supplementary Material). The isogenicity between each deleted strain was confirmed by pulsed-field gel electrophoresis (see Figure S2 in Supplementary Material).

### Peripheral Blood Mononuclear Cell (PBMC) from Healthy Donors, Human NK Cells Isolation, and NK92 Human Cell Line

– PBMCs (Cryopreserved Human Peripheral Blood Mononuclear Cells) were isolated from heparinized blood of healthy volunteers by gradient centrifugation on Ficoll-Hypaque (Lymphoprep, Norway). PBMCs were unfrozen and then kept in IL-2 overnight (100 UI/ml). After cell sorting, NK cells were immediately resuspended in IL-2 supplemented medium and then infected. All donors were recruited at the blood transfusion center (Nantes, France). Informed consent was obtained from all individuals and all experiments were approved by the Ethics Committee of Tours, France (2015-DC-1) (Biocollection Authorization Number DC-2014-2340), and performed in accordance with relevant guidelines and regulations.
– Human NK cells were sorted from PBMC of healthy donors with Untouch NK cell isolation kit (Miltenyi Bitoec). CD56^bright^ and CD56^dim^ NK cells were isolated from PBMC of healthy donors by Flow Cytometry Cell Sorting using CD56^pos^ and CD3^neg^ gating routinely yielded cell population with purity of 95% (FACSARIA cell sorter, BD Bioscoences). Isolated NK cells were then cultured in medium supplemented with 100 U/ml IL-2 (Proleukin, Aldesleukin, Chiron).
– NK92 is an IL-2-dependent human tumor NK cell line CD56^bright^ CD3^neg^, expressing neither the killer cell immunoglobulin-like receptor (KIR^neg^) nor CD16 (see Figure S3 in Supplementary Material).

### Infection

– PBMC, sorted human NK cell, or NK92 cell lines were cultured at 37°C in 5% CO_2_ in RPMI 1640 medium (Gibco) containing glutamine (Gibco) with 10% fetal bovine serum (Gibco, <10 EU/ml endotoxin contamination), penicillin-streptomycin (PS), and 100 U/ml IL-2 (Proleukin, Aldesleukin, Chiron) (13). Cells were seeded in 96-well plates (250,000 per well in 200 μl).
– PA strains were grown overnight in Brain Heart Infusion medium at 37°C. Bacterial inoculum was calibrated by nephelometry. Cells and bacteria were cocultured with a 1:1 bacteria to NK cell ratio. In PBMC, we also applied a 1:1 bacteria to NK cell ratio, assuming 10% NK cells among PBMC. After 2 h of coculture in PS-free RPMI and IL-2 supplemented medium, the wells were centrifuged at 1,500 RPM for 2 min and placed in fresh IL-2 supplemented RPMI medium with PS to prevent bacteria overgrowth until the 24th hour. Non-infected wells were similarly centrifuged and resuspended in fresh medium supplemented with IL-2 and penicillin/streptomycin. During infection the medium was also supplemented with IL-2 to ensure continuous stimulation all along experiments. When mentioned, the medium was supplemented with IL-12 (Miltenyi) at a concentration of 5 ng/ml for the first 2 h. In some conditions, transparent PET membranes (filter with 0.4 µm pore size) were used in culture wells to prevent NK-bacteria contact.

### Kinase Study

MEK/ERK kinase inhibitor (PD98059) were purchased from Sigma Aldrich (France). Inhibitor was diluted according to the manufacturer instructions with dimethyl sulfoxide (DMSO) at 0.04%. Before the PA challenge, NK cells were incubated for 1 h at 37°C with 10 µM of inhibitor (14). Cultures with DMSO 0.04% under inhibitor-free conditions were also prepared to control its potential effect on cytokine response.

### Pneumonia Model

We used our validated pneumonia model (4, 15, 16). Six-week-old female Swiss mice (20–24 g) were anesthetized with isoflurane. A 24-G transtracheal feeding needle was inserted to inject 75 µl of bacterial suspension adjusted to 10^8^ CFU/ml. The same anesthesia procedure and treatment with 75 µl of saline buffer was applied to control mice (SHAM). Mice were maintained on a 12-h light/dark cycle. All experimental protocols were approved by the Committee of Animal Ethics of the Pays de Loire (CEEA-2012-233) and all methods were carried out in accordance with the guidelines and regulations.

### Cell Labeling

Antibodies were purchased from BD Biosciences unless otherwise stated. Data were collected with four-color FACSCalibur (BD Biosciences) and LSRII cytometer (Benton Dickinson, Le Pont de Claix, France) and analyzed using FlowJo 6.2 software (Ashland, OR, USA). For PBMCs, NK cell gating was performed with anti-CD56-APC (NCAM16.2, #341026), anti-CD3-PerCP (SK7, #345766), and the corresponding isotype-matched control mAb. Cytolytic activity (CD107a membrane expression) was assessed with CD107-FITC (H4A3, #555800) after 5 h of *in vitro* incubation. To study IFN-γ production, the cells were treated with Brefeldin A (Sigma) at 10 µg/ml for 5 h. IFN-γ intracellular staining was then performed with anti-IFN-γ-PE (B27, #554701) after cell permeabilization with PFA 4% (Sigma) at 4°C overnight followed by Saponin 0.1% (Sigma). Cell viability among NK cell line was assessed by APC-Fixable Viability Dye Kit eFluor 780 staining (eBioscience).

In mouse pneumonia model, cell suspensions were obtained by mechanical and collagenase D digestion (1 h at 37°C) of lungs collected 24 h postinfection. NK cell gating was performed with anti-NK1.1-BV 421 (#562921) and anti-CD3-APC (#553066). For IFN-γ intracellular staining, after red blood cells lysis (RBC lysis buffer, Ozyme), 70 µm filtered cells were cultured 5 h in RPMI 1640 medium supplemented with 2% FCS with GolgiPlug, washed twice, and then stained for surface markers. Fixation and permeabilization was performed following manufacturer instructions (BD Cytofix/Cytoperm kit, BD Bioscience). Anti-IFN-γ AlexaFluor 488 (#557724) antibody or its rat IgG1κ isotype control were incubated overnight at 4°C. Cells were washed twice before analysis on a LSRII flow cytometer (BD Bioscience).

For confocal microscopy, cells were stained with primary rabbit anti-human NCR2 antibody (#133668, Abcam) for 30 min at 4°C (1/100) and secondary goat anti-rabbit Alexa 568 antibody (#11011, Life Technologies) for 20 min at 4°C (1/400). NK92 cells were seeded onto glass cover-slips with 2-octyl Cyanoacrylate DERMABOND™, Ethicon, and underwent 2 min of centrifugation at 1,500 rpm. Infection was performed with PA-GFP immediately before confocal visualization (Nikon A1 RSi) with Plan APO 60× objective with a numerical aperture of 1.40. Stack acquisition was performed at 30-s intervals, scan size was 512 × 512, 5× zoom, pinhole 2 (Airy unit), and 2 µm step sizes. Images were not processed after acquisition.

### Cytokine Quantification by Enzyme-Linked Immunosorbent Assay (ELISA)

All ELISA kits were purchased from eBioscience.

– CD56^bright^, CD56^dim^ NK cells, and NK92 cell lines: IFN-γ production was quantified in cell-free culture supernatant after 24 h of culture.
– Mice lungs: immediately after removal, lungs were mechanically homogenized in phosphate-buffered saline (PBS, pH 7.4), 0.1% Triton X-100 containing 1 mM protease inhibitor cocktail (Sigma). IFN-γ concentration was determined and normalized on protein concentration (BCA protein assay kit, Rockford, IL, USA) (16).

### RT-PCR Analysis

Total RNA was isolated using the RNeasy kit (Qiagen) and treated for 45 min at 37°C with DNase (Promega). RNA (1 µg) was reverse-transcribed with superscript III reverse transcriptase (Life Technologies). The cDNA was subjected to RT-qPCR in a Bio-Rad iCycler iQ system using the QuantiTect SYBR Green PCR kit (Qiagen). See primer sequences in the online supplementary table (see Table S4 in Supplementary Material). Relative gene expression was normalized on GAPDH and calculated using the 2^−ΔΔCt^ method with samples from the IL-12 free, non-infected group as calibrators.

### Western Blotting

Cell pellets were suspended in cold RIPA buffer, protease inhibitor, and 1% phosphatase cocktails (Sigma), mixed with the SDS sample buffer, boiled, and separated by SDS-PAGE (10% TGX Precast Gel, Bio-Rad). Proteins were transferred onto a Trans-Blot^®^ Turbo™ membrane. Membranes were successively probed with primary antibodies, DyLight™ 680 or 800 secondary antibodies and revealed on an infrared imager (LICOR ODYSSEY). The following primary antibodies were used (cell signaling unless otherwise stated): anti-Phospho-Stat4 (Tyr693) (D2E4) rabbit mAb, anti-Phospho-p44/42 MAPK (Erk1/2) (9101S) rabbit mAb, anti-p44/42 MAPK (Erk1/2) (9102S) rabbit mAb or anti-Actin (A5441) mouse mAb (Sigma).

### Statistical Analysis

Statistical analyses were performed with GraphPad prism software (La Jolla, CA, USA). Continuous non-parametric variables were expressed as the median (25th to 75th percentile). The Kruskal–Wallis test was used to compare multiple groups. The *post hoc* Dunn’s test was used to perform multiple comparisons. Survival curves were compared to a log-rank test. *P* < 0.05 was considered to be statistically significant.

### Data Availability

The datasets generated and/or analyzed during the current study are available from the corresponding author on request.

## Results

### CD56^bright^ NK Cells Are the Main Source of IFN-γ Production after PA infection

**Figure 1:**
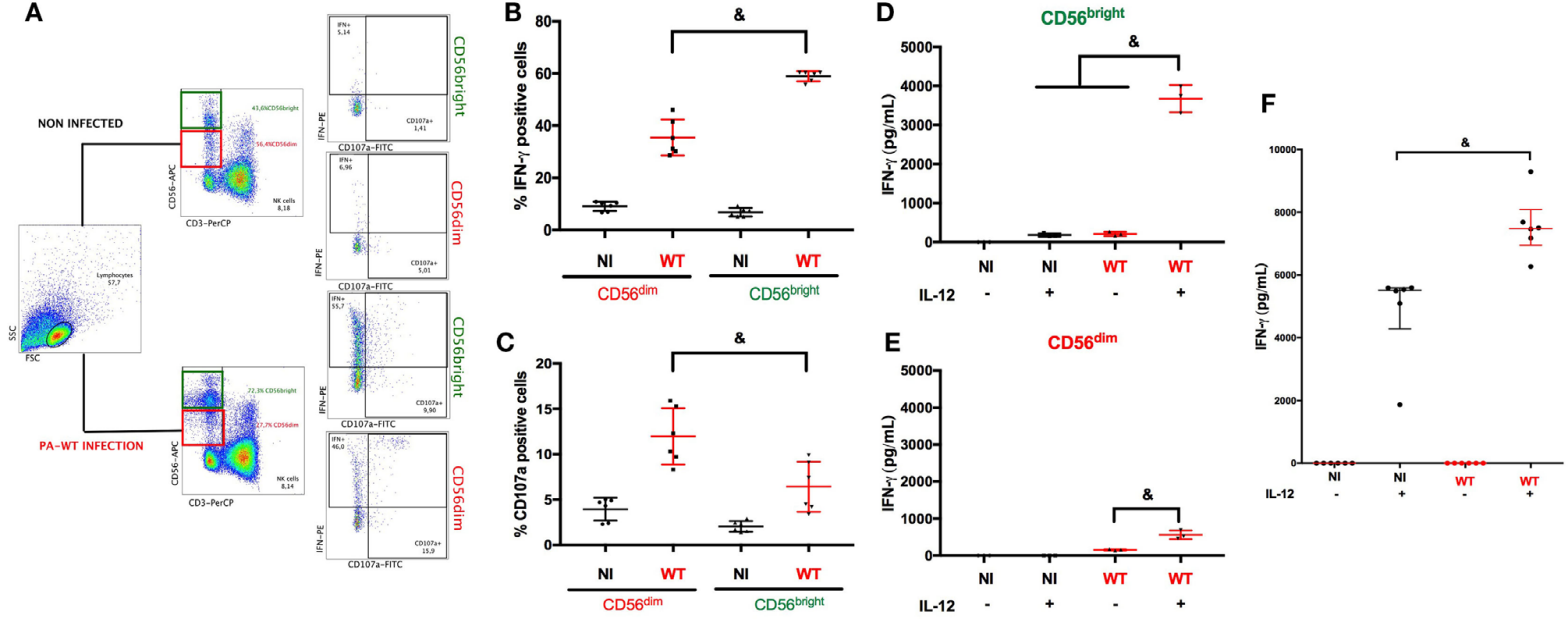
Natural killer (NK) cells IFN-γ degranulation response after PA challenge with or without IL-12 priming. Cytokine (IFN-γ) and cytolytic (CD107a) activity of NK cells among PBMC were assessed with or without PA-WT infection **(A–C)**. Representative density plots illustrating intracellular IFN-γ and CD107a expression in CD3^neg^ CD56^bright^ or CD3^neg^ CD56^dim^ among CD3^neg^ CD56^pos^ cells in lymphocyte gate by flow cytometry **(A)**. Histograms of CD3^neg^ CD56^dim^ and CD3^neg^ CD56^bright^ IFN-γ^+^ **(B)** or CD107a^+^ **(C)** in NI or PA-WT-infected conditions (Representative of 6 healthy donors, 2 distinct experiments, with 3 different donors per experiments). IFN-γ concentration was measured (ELISA) in supernatant of CD56^bright^ **(D)** and CD56^dim^ **(E)** sorted from human PBMC (representative of three different donors) or NK 92 cells **(F)** (six distinct experiments) after a 24-h infection with or without IL-12 stimulation. Data are shown as the median and interquartile range. ^&^*p* < 0.05, PBMC, peripheral blood mononuclear cell; NI, non-infected, WT, PA-WT infection.

The close interaction between NK cells and PA has been reported previously (4, 17, 18). We first focused on the determinant of IFN-γ production by NK cells following PA-infection. We performed PA infection in PBMC to assess NK cells response in a physiological microenvironment. NK cells are heterogeneous with different subsets specialized in either cytokine or cytotoxic activities (8), thus we aimed to select the main subset of NK cells specialized in IFN-γ production. After gating on CD56^pos^ and CD3^neg^ cells, we analyzed intracellular IFN-γ staining and CD107a membrane expression (surrogate marker of degranulation) among CD56^bright^ and CD56^dim^ subsets (Figure 1A). After PA-WT infection, the proportion of CD56^bright^ NK cells increased and exhibited higher IFN-γ activity and lower cytotoxic activity compared to the CD56^dim^ subset (Figures 1B,C). To confirm preferential IFN-γ activity of CD56^bright^ subset, we sorted NK cells from PBMC by cytometry according to their subset (CD56^bright^ or CD56^dim^) and subsequently 24-h infected each subset with or without IL-12 stimulation. IL-12 produced by dendritic cells (DC) is critical for NK cell activation (19). As previously described, without IL-12 priming, NK cells produced low levels of IFN-γ in response to PA. After IL-12 stimulation, PA significantly increased IFN-γ as compared to non-infected cells. Moreover, CD56^bright^ NK cells produced higher level of IFN-γ than CD56^dim^, inciting us to study CD56^bright^ subset to precise intracellular pathways leading to IFN-γ production (Figures 1D,E).

To further explore microenvironment influenced on IFN-γ response after PA infection, we used a human NK cells line (NK92) specialized in cytokine production and sharing the CD56^bright^ NK cells receptor repertory (CD56^bright^ KIR^neg^ CD16^neg^) (8) (see detailed phenotype of NK92 in supplemental Figure S3). Similarly to sorted human NK cells, without IL-12 priming, NK92 cells released low level of IFN-γ after PA infection (Figure 1F). IL-12 triggered IFN-γ production and PA further increased IFN-γ level after IL-12 priming. As compared to IL-15 or IL-21, also reported to participate in cytokine response of NK cells, IL-12 stimulation gave rise to higher IFN-γ production (see Figure S5 in Supplementary Material).

### PA Increases IFN-γ Production in a STAT-4-Independent Pathway

**Figure 2:**
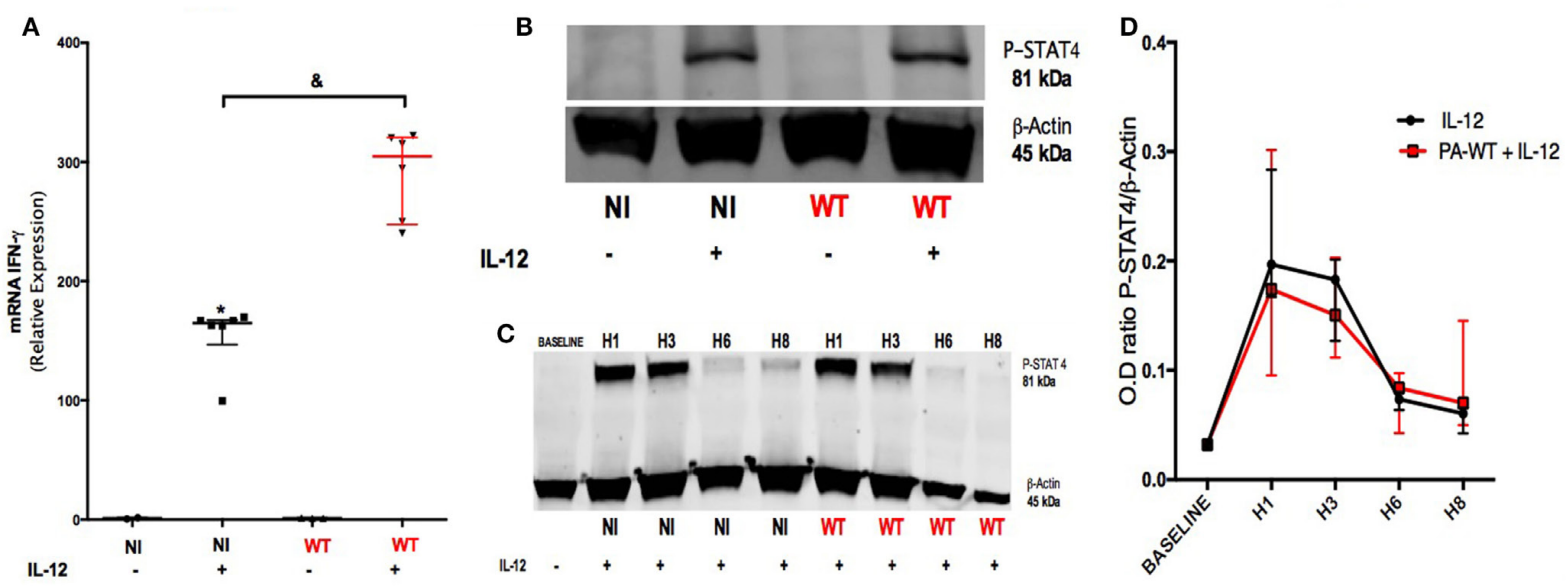
Pathways leading to IFN-γ production in IL-12-treated natural killer cells with or without PA-WT infection. IFN-γ mRNA **(A)** was measured by RT-PCR in NK92 cell after a 6-h culture (expressed as relative expression compared to GAPDH). **(B)** Western Blot membrane of phospho-STAT-4 after a 2-h culture in non-infected (NI) or PA-WT infected (WT) NK92 with or without IL-12 priming. **(C)** Time course over 8 h of STAT-4 phosphorylation in NI or WT conditions in NK92. **(D)** Optical density (OD) of P-STAT4/β-Actin ratio evolution from baseline over 8 h after infection with IL-12 stimulation. Western Blot membranes **(B,C)** were cropped to improve the clarity and conciseness of the presentation, see the full-length membranes in Figure S7 in Supplementary Material. Data are representative of six **(A)** and three **(B–D)** independent experiments. STAT-4, signal transducer and activator of transcription-4; DMSO, dimethyl sulfoxide; NI, non-infected NK92 cells. ^&^*p* < 0.05.

Our objective was to identify the pathways involved in IFN-γ production after IL-12 stimulation and the influence of PA infection on these pathways. For this purpose, PCR analysis was performed on NK92 cell line after PA-WT infection with or without IL-12 stimulation. The protein and mRNA IFN-γ followed the same trends (Figures 1F and 2A) in IL-12-treated NK 92 cells infected with PA, suggesting that PA infection regulates IFN-γ at a pre-transcriptional level. STAT-4 is the main transcriptional factor involved in both IL-12 receptor (IL-12R) signaling and IFN-γ mRNA transcriptional activity (20). Thus, we compared the activation of STAT-4 after 2-h infection with or without IL-12 stimulation in NK92 cell line. Compared to IL-12-treated NK cells, PA did not affect the phosphorylation of STAT-4, which is induced by IL-12 treatment (Figures 2B–D). We concluded that PA could modulate the production of IFN-γ in IL-12-treated NK cells through a pathway independent from IL-12R.

### T3SS and Its Effector Modulate IFN-γ Production after Direct PA-NK Binding

**Figure 3:**
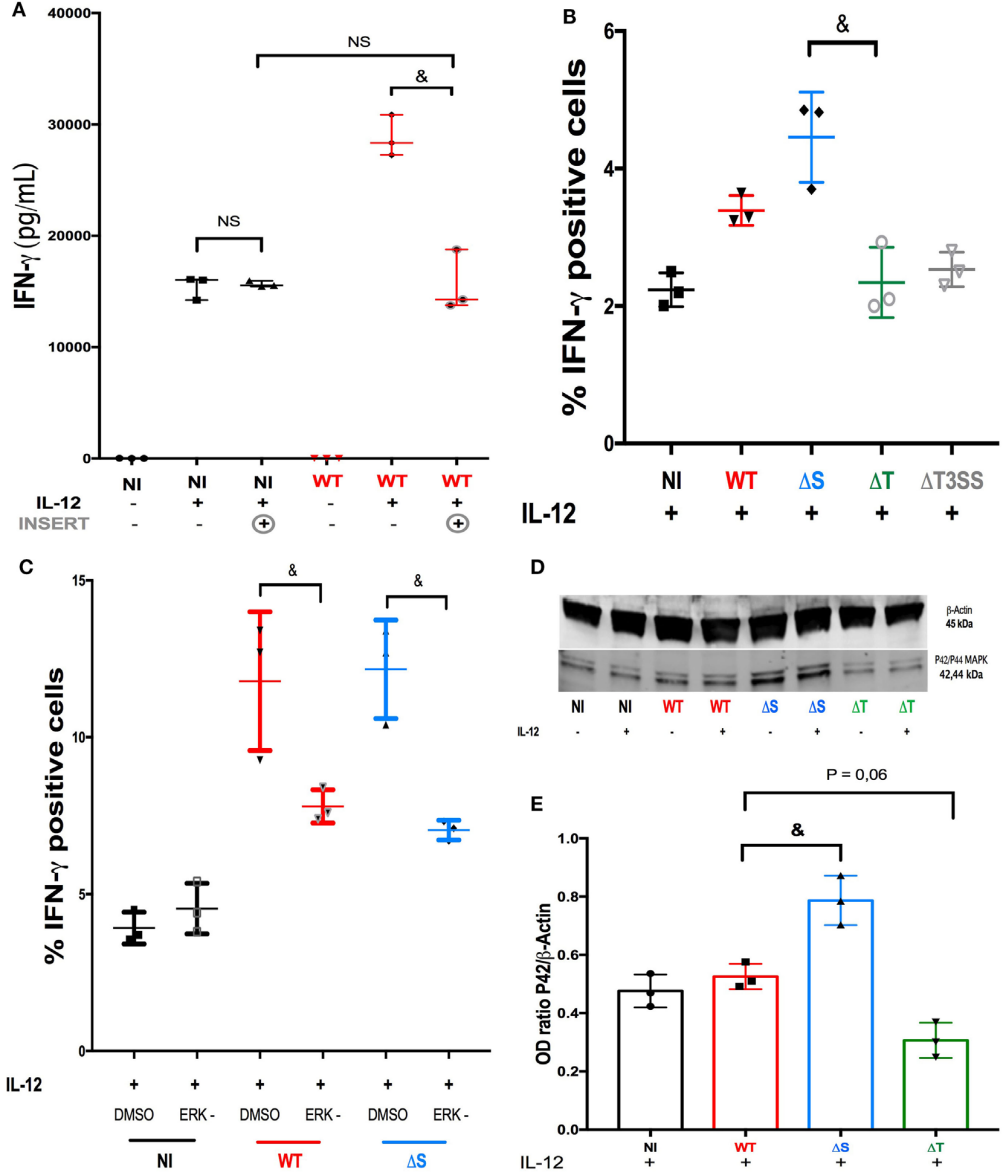
ERK is a key regulator of IFN-γ activity after ExoT stimulation. **(A)** To determine the role of bacteria-to-NK cells direct binding on cytokine production, IFN-γ concentration was measured (ELISA) in the supernatant of non-infected (NI) or PA-WT infected (WT) NK92 cells with or without IL-12 priming and with or without an insert (three distinct experiment). Histograms representative of IFN-γ^+^ cells among sorted human NK cells in NI or PA infected conditions after IL-12 stimulation **(B)** with or without ERK inhibitor **(C)** (representative of three distinct experiments with one different healthy donor per experiment). Western Blot membrane of p42/p44 (ERK1/2) phosphorylation **(D)** and corresponding optical density (OD) (p42 MAPK/β-Actin ratio) **(E)** analysis after a 2-h culture in NI, PA-WT, PA-ΔS, or PA-ΔT infected conditions in NK92 cells with or without IL-12 priming. Western Blot membrane **(D)** was cropped to improve the clarity and conciseness of the presentation, see the full-length membranes in Figure S7 in Supplementary Material (Representative of three distinct experiments). Data are presented as the median and interquartile range. ^&^*p* < 0.05; NS, Non significant difference; DMSO, dimethyl sulfoxide; ERK, ERK inhibition; NI, Non-infected NK cells; WT, PA-WT infection (expressing ExoS, T, and Y); ΔS, PA deleted in ExoS (expressing ExoT and Y); ΔT, PA deleted in ExoT (expressing ExoS and Y); ΔT3SS, PA deleted in needle complex (expressing ExoS, T, and Y). MEK, mitogen-activated protein kinase; ERK, extracellular-signal regulated kinase (ERK-1/2).

Live confocal microscopy recorded immediately after PA-GFP infection suggested direct bacteria-to-cell contact (Video S6 in Supplementary Material). When NK92 cells were cultured under a filter (preventing any direct contact with PA), the infection failed to increase the production of IFN-γ in IL-12-treated NK cells (Figure 3A). We concluded that direct bacteria-to-cell contact was involved in the cytokine activity modulation. During infection, PA uses a complex T3SS to inject effector proteins (Exoenzymes S, T, and Y) into host cells (21), these proteins interfere with the intracellular signaling pathways (22), the function and viability of target cells (21). Exoenzymes effects on the cytokine response in NK cells had not been investigated to date. For this purpose, sorted human NK cells were challenged with three PA isogenic strains deleted for T3SS or its effectors and compared to PA-WT (Figure 3B) (see Isotype control for intracellular in Supplemental Figure S8). The exoenzymes deletion did not modify bacterial growth (Figure S9 in Supplementary Material) or the survival of NK cells during infection (Figure S10 in Supplementary Material). Needle complex (PA-ΔT3SS) deletion reduced IFN-γ production in IL-12-treated NK cells as compared to PA-WT infection. Contrary to PA-ΔS (expressing ExoT), infection with a strain lacking ExoT (PA-ΔT) decreased IFN-γ activity as needle complex deletion did, suggesting that ExoT is a determinant of IFN-γ activity in NK cells. These results demonstrate that the production of IFN-γ by NK cells is not solely driven by IL-12 stimulation, but that exoenzymes can also modulate cytokine production.

### MEK/ERK Pathways Is Involved in ExoT-Induced IFN-γ Activity after PA Infection

*Pseudomonas aeruginosa* exoenzymes were already reported to interfere with Ras family proteins which control ERK phosphorylation (23). In view of the singular role of ExoT (Figure 3B), the next step was to investigate the pathway(s) involved in ExoT-induced IFN-γ production under IL-12 stimulation. Thus, we studied intracellular IFN-γ staining in sorted human NK cells after either PA-WT (full set of exoenzymes) or PA-ΔS (expressing ExoT but not ExoS) infection (Figure 3C) with or without ERK inhibitor. ERK inhibitor induced a major reduction in PA-ΔS and PA-WT whereas did not affect the percentage of IFN-γ^+^ cells in NI condition after IL-12 stimulation. Cell viability study after infection with PA-WT or deleted strains with or without ERK inhibitor ensured that cells mortality did not explain these differences (see Figure S10 in Supplementary Material). Western Blot experiments in NK92 cell line (Figures 3D,E) confirmed ExoT-dependent ERK phosphorylation after 2-h infection (see the membrane with unphosphoryled form of ERK in Figure S7 in Supplementary Material). Comparable phosphorylation of ERK with or without IL-12 suggest that PA may activate NK cells through ERK phosphorylation independently of IL-12 stimulation but that IL-12 priming remains a prerequisite for IFN-γ activity. As a result, in our model, ERK is specifically involved in IFN-γ production after PA infection but not in IL-12 dependent IFN-γ production.

### The T3SS Is Involved in Mouse Mortality in the PA Pneumonia Model and Influences IFN-γ Levels in Lungs

**Figure 4:**
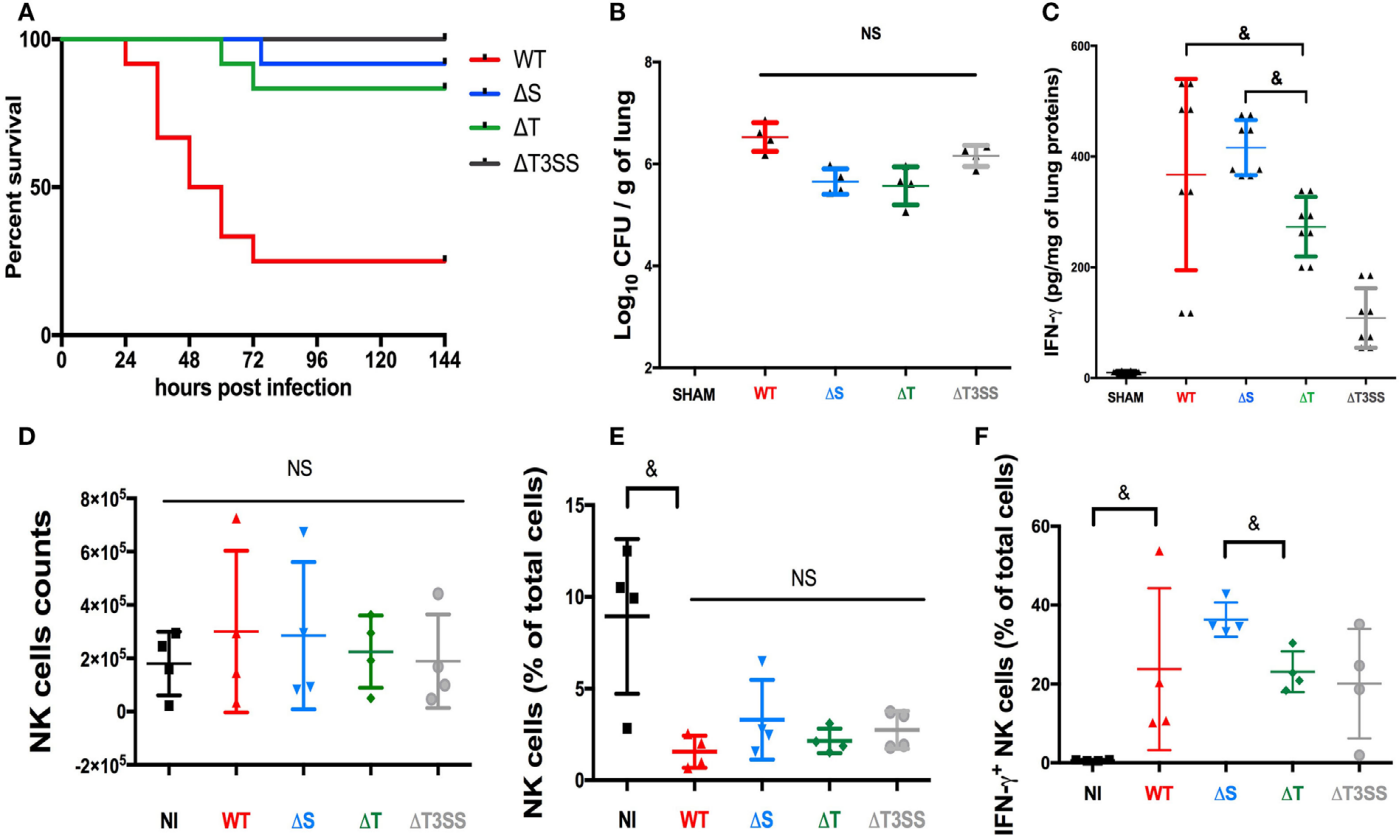
Involvement of the type III secretion system (T3SS) in a mouse PA pneumonia model. **(A)** 6 days (144 h) survival after PA-WT pneumonia compared to three deleted strains (PA-ΔS, PA-ΔT, and PA-ΔT3SS); bacterial count **(B)** and IFN-γ **(C)** levels in lungs 24 h after infection. SHAM Swiss mice were instilled with 75 μl of saline buffer (representative of 2 independent experiments with 2 and 4 mice for each strain, respectively for the bacterial count and cytokine level). Histograms after cytometry analysis representing NK cells absolute number **(D)**, NK cells percentage among whole lung cells suspension **(E)**, and the percentage after IFN-γ^+^ NK cells **(F)** (two independent experiments with two mice in each group). Data are presented as the median and interquartile range. ^&^*p* < 0.05, NS, non-significant difference; CFU, colony-forming unit.

We have already reported the critical role of NK cells in controlling infection and producing IFN-γ in a lethal mouse PA-pneumonia model (4). Using the same model, we assessed the role of T3SS and its effectors on mouse mortality and IFN-γ production in mice lungs. The deletion of T3SS or its effectors reduced the mortality rate in infected mice (Figure 4A) irrespective of the bacterial load in lungs 24 h after infection (Figure 4B). These data demonstrate *in vivo* the critical role of T3SS and its effectors. The IFN-γ level in lungs followed the same trends as observed in our *in vitro* model, with significantly higher IFN-γ activity in PA-expressing ExoT (PA-WT and PA-ΔS) than in PA-ΔT pneumonia (Figure 4C). Cytometry analysis in lungs after PA pneumonia confirmed that IFN-γ is mainly produced by NK cells (see Figure S11 in Supplementary Material). After PA pneumonia, the absolute number of NK cells was not different compared to sham condition (Figure 4D) but the percentage of NK cells was reduced (Figure 4E). There was no difference between PA-WT and deleted strains regarding NK cells percentage. PA-ΔS (Expressing ExoT) led to higher percentage of IFN-γ^+^ NK cells as compared to PA-ΔT (Figure 4F). These data confirmed the key role of ExoT *in vivo* on IFN-γ activity modulation.

## Discussion

While the treatment of NK cells by myeloid-derived cytokines (such as IL-12) is required for the production of IFN-γ, our results demonstrate that PA can directly alter IFN-γ production *via* the modulation of ERK through exoenzyme injection in NK cells. Our findings were supported and validated *in vivo* in a murine model of PA-pneumonia. IFN-γ was already documented to enhance the synthesis of virulence factor of PA. Thus, the control of NK cells IFN-γ activity by PA through Exoenzyme infection is a major concept (7).

*Pseudomonas aeruginosa* infection leads to an IFN-γ response that usually promotes major histocompatibility complex I and II molecule upregulation, and macrophage and CD4^+^ T cell activation (24). Even if inflammation is central to eliminate the pathogen in the early phase of the infection, an uncontrolled inflammatory response could lead to tissue damage, organ dysfunction, and increase the risk of further infection (25). PA has been previously reported to be capable of taking advantage of the IFN-γ response to enhance the synthesis of its virulence factors (7, 26). On the other hand, in PA-ocular infection in IL-12 knockout animals, IFN-γ reduction also resulted in unchecked bacterial growth and perforation (10).

IL-12 is the main actor in NK/DC cross talk. We have already demonstrated that in patients highly susceptible to infection, such as patients with brain injuries, IL-12 is able to restore IFN-γ production *ex vivo* in NK cells (27). Here, we confirmed the key role of IL-12/STAT4 engagement in the IFN-γ response to PA infection, specifically in CD56^bright^ NK cells (20). These data are supported by a preferential lymph node localization of CD56^bright^ NK cells, where IL-12 stimulation through NK/DC cross talk prevails (8).

We have demonstrated that a direct bacteria-to-cell contact was required to give rise to a cytokine response (Figure 3A). The hypothesis of PAMP recognition by NK cells through toll-like receptors has already been explored, but TLR blocking did not suppress IFN-γ response (28) suggesting alternative recognition pathways. Thus, we hypothesized that PA could release mediators directly into NK cytosol and modulate host response. Among the large arsenal of PA virulence factors, the needle complex (T3SS) allows the injection of three effectors (ExoS, T, Y) into the cytoplasm of the host cell. In a clinical setting, T3SS expression is correlated with poor outcomes in pneumonia in Intensive care Unit (29). Here, we found a pivotal role of T3SS in IFN-γ production. In particular, ExoT (expressed by more than 95% of PA strains (30)) stood out as the main trigger of IFN-γ production. These data we confirmed *in vivo*. ERK involvement in PA pathogenicity has already been reported previously as an internalization pathway for the bacteria (31). In our model, NK cells infection with PA expressing ExoT increased IFN-γ production and ERK phosphorylation (Figures 3C,D). The important gap between the percentage of IFN-γ-positive NK cells among PBMC and sorted NK cells (Figures 1A and 3B,D) underscores the key role of the microenvironment to initiate inflammatory response after PA infection. This is confirmed in our PA-pneumonia mice model (Figure 4F).

Given our results and the previous description of ExoT and ExoS functions *in vivo*, we have tried to envision how PA infection might modulate the cytokine response and ERK phosphorylation. ExoT and ExoS are bifunctional toxins with N-terminal Rho GTPase-activating protein (GAP) domains, and C-terminally encoded ADP ribosyltransferase (ADPRT) domains. ExoT and ExoS GAP domains have been reported to induce an actin cytoskeleton rearrangement leading to apoptosis. The ADPRT domain of ExoT interacts with the Crk protein (32), which binds to Cbl-b (E3 ubiquitin ligases) and undergoes rapid proteasomal degradation (33). In non-infected conditions, Cbl-b downregulates ERK phosphorylation (34, 35) and IFN-γ production (36). We can hypothesize that during PA-infection, ExoT binding to Cbl-b suppresses ERK regulation and increases IFN-γ production. Conversely, the ADPRT domain of ExoS inactivates cytoskeletal regulators, such as Ras family proteins, which can compromise ERK phosphorylation (23).

Our study presents limitations. NK92 exhibit a highly specific receptors repertory, which was not assessed in sorted NK cells, especially regarding KIR expression. Thus, the parallel with sorted human NK cells have to be tempered. siRNA or knockout cell lines would have discard the off-target effects of kinase inhibitor and increase specificity of IFN-γ pathway study. Exoenzyme detection in host cell cytoplasm after PA infection could confirm ExoT involvement in IFN-γ production. Complemented strains usually ensure a higher level of isogenicity as compared to deleted strains. Nevertheless, the constant bacterial load in the lungs of infected mice and the comparable generation time for all strains ensured that deletion did not alter bacterial growth. Finally, although PA-WT and deleted strains exhibit comparable growth, mice displayed enhanced survival when infected with PA-deleted strains as compared to PA-WT in our model. These data suggest that although each Exoenzyme triggers a singular host function, PA pathogenicity results in combined effect of the whole virulence factor apparatus.

In conclusion, without IL-12 priming, PA escapes recognition by NK cells, preventing any cytokine response. PA infection enhances IFN-γ production by NK cells through T3SS and its effectors especially ExoT. Poor outcome in PA pneumonia with strains expressing T3SS (29) could be explained by an exacerbated inflammatory response mediated by ExoT. Last, our data are in line with the current clinical and experimental research that suggests targeting T3SS or exoenzymes during PA infections (37, 38).

## Ethics Statement

For PBMC: all donors were recruited at the blood transfusion center (Nantes, France). Informed consent was obtained from all individuals and all experiments were approved by the Ethics Committee of Tours, France (2015-DC-1) (Biocollection Authorization Number DC-2014-2340), and performed in accordance with relevant guidelines and regulations. For mice: mice were maintained on a 12-h light/dark cycle. All experimental protocols were approved by the Committee of Animal Ethics of the Pays de Loire (CEEA-2012-233), and all methods were carried out in accordance with the guidelines and regulations.

## Author Contributions

MV and KA designed all the experiments. MV, AR, GD, AB, CJ, JC, CR, and KA wrote the main manuscript text. All authors reviewed the manuscript. MV and AR participated equally. PH performed and analyzed confocal microscopy pictures. MV, AR, AB, CJ, and GD performed the experiments.

## Conflict of Interest Statement

The authors declare that the research was conducted in the absence of any commercial or financial relationships that could be construed as a potential conflict of interest. The reviewer BS and handling editor declared their shared affiliation.
