# Supplementary material for: Exoenzyme T Plays a Pivotal Role in the IFN-γ Production after Pseudomonas Challenge in IL-12 Primed Natural Killer Cells
Source: Front Immunol. 2017 Oct 10;8:1283. doi: 10.3389/fimmu.2017.01283 (PMC5641345; doi:10.3389/fimmu.2017.01283)
Supplement: Supplementary file 2 [file Presentation_1.pdf]

TITLE PAGE: Exoenzyme T plays a pivotal role in the IFN-γ production after *Pseudomonas* challenge in IL-12 primed natural killer cells.

Mickael Vourc'h, MD (1,2) [#]; Antoine Roquilly MD, PhD (1,2) [#]; Alexis Broquet, PhD (1); Gaelle David (3,4); Philippe Hulin; (5) Cedric Jacqueline, PhD (1); Jocelyne Caillon PharmD, PhD (1); Christelle Retiere PhD (3,4); Karim Asehnoune*, MD, PhD (1,2,6).

Affiliations

1. Laboratoire UPRES EA3826 « Thérapeutiques cliniques et expérimentales des infections », IRS2 - Nantes Biotech, Université de Nantes, Nantes, France.
2. Intensive Care Unit, Anesthesia and Critical Care Department, Hôtel Dieu, University Hospital of Nantes, Nantes, France.
3. Etablissement Français du Sang, Nantes, France.
4. CRCINA, INSERM U1232, CNRS, Université d'Angers, Université de Nantes, France.
5. MicroPICell, Cell and Tissue Imaging Core, UMS Inserm 016/CNRS 3356/FED 4203.
6. Corresponding author: Karim Asehnoune, MD, PhD. Intensive Care Unit, Anesthesia and Critical Care Department, Hôtel Dieu, University Hospital of Nantes, Nantes, France, F-44093, France, Karim.asehnoune@chu-nantes.fr +33 (0)2 40 08 33 33.

[#] These 2 authors participated equally

* Corresponding author

Online-only Material: Supplementary Figures, Video, Table and Legends

Supplementary Figure S1: PCR analysis of exoenzymes or PscC expression in wild-type and deleted strains

Supplementary Figure S2: Pulse-field gel electrophoresis of the wild-type, deleted and GFP strains

Supplementary Figure S3: Detailed phenotype of NK 92 cell line (Cytometry analysis)

Supplementary Table S4: PCR primers

Supplementary Figure S5: Influence of IL-12, IL-15 and IL-21 on IFN-g response after PA-WT infection

Supplementary Video S6: Live NK-PA-WT GFP interaction

Supplementary Figure S7: Full-length Western Blot gels from Figure 2B,C and 3D

Supplementary Figure S8: IgG1 Isotype control profile for intracellular IFN-γ staining

Supplementary Figure S9: Growth curves of PA-WT and deleted strains

Supplementary Figure S10: Mortality after PA infection among sorted NK cells

Supplementary Figure S11: IFN-g source in lungs after murine PA-pneumonia.

Supplementary Figure S1

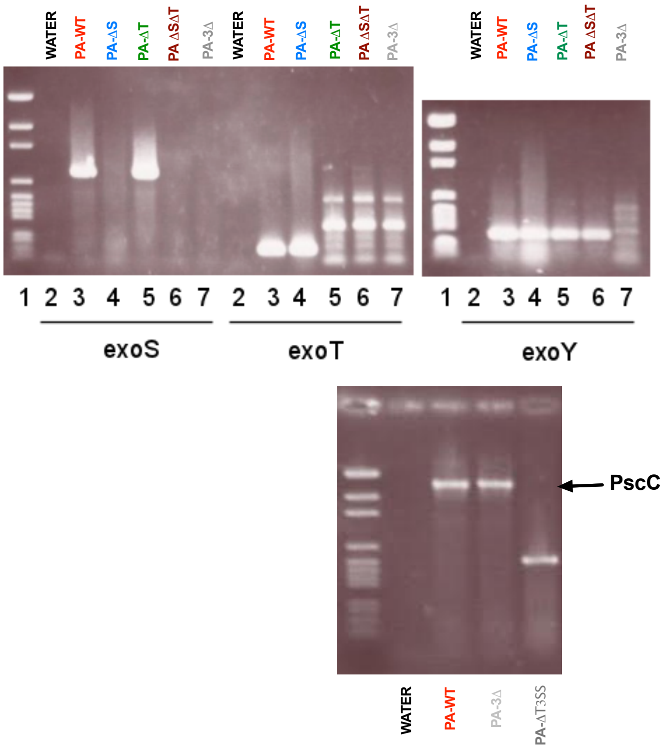

<u>Title Figure S1</u>: PCR analysis of exoenzymes or PscC expression in wild-type and deleted strains

<u>Legend Supplementary Figure S1</u>: Exo: Exoenzyme, **PA-WT**: PA wild-type strain expressing ExoS, T, and Y, **PA-ΔT**: PA deleted in ExoT, **PA-ΔS**: PA deleted in ExoS, **PA-ΔT3SS**: PA deleted in needle complex but expressing ExoS, T, and Y, **PA-3Δ:** PA deleted in ExoS, T and Y, **PA-ΔSΔT**: PA deleted in ExoS and T, **PscC**; Protein secretion system.

Supplementary Figure S2

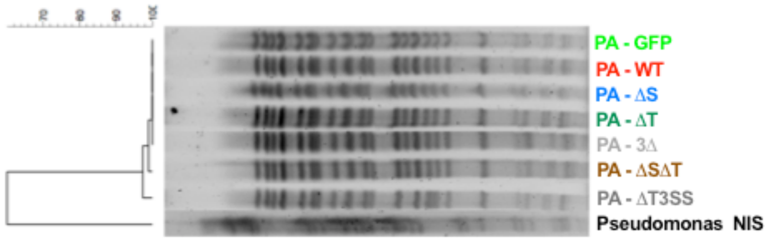

<u>Title Figure S2</u>: Pulse-field gel electrophoresis of the wild-type, deleted and GFP strains

<u>Legend Supplementary Figure S2</u>: NIS: Non-isogenic PA strain, **PA-WT-GFP**: PA wild-type strain expressing ExoS, T, Y, and the Green fluorescent protein (GFP), **PA-WT**: PA wild-type strain expressing ExoS, T, and Y, **PA-ΔT**: PA deleted in ExoT, **PA-ΔS**: PA deleted in ExoS, **PA-ΔT3SS**: PA deleted in needle complex but expressing ExoS, T and Y, **PA-3Δ:** PA deleted in ExoS, T, Y, **PA-ΔSΔT**: PA deleted in ExoS and T.

Supplementary Figure S3

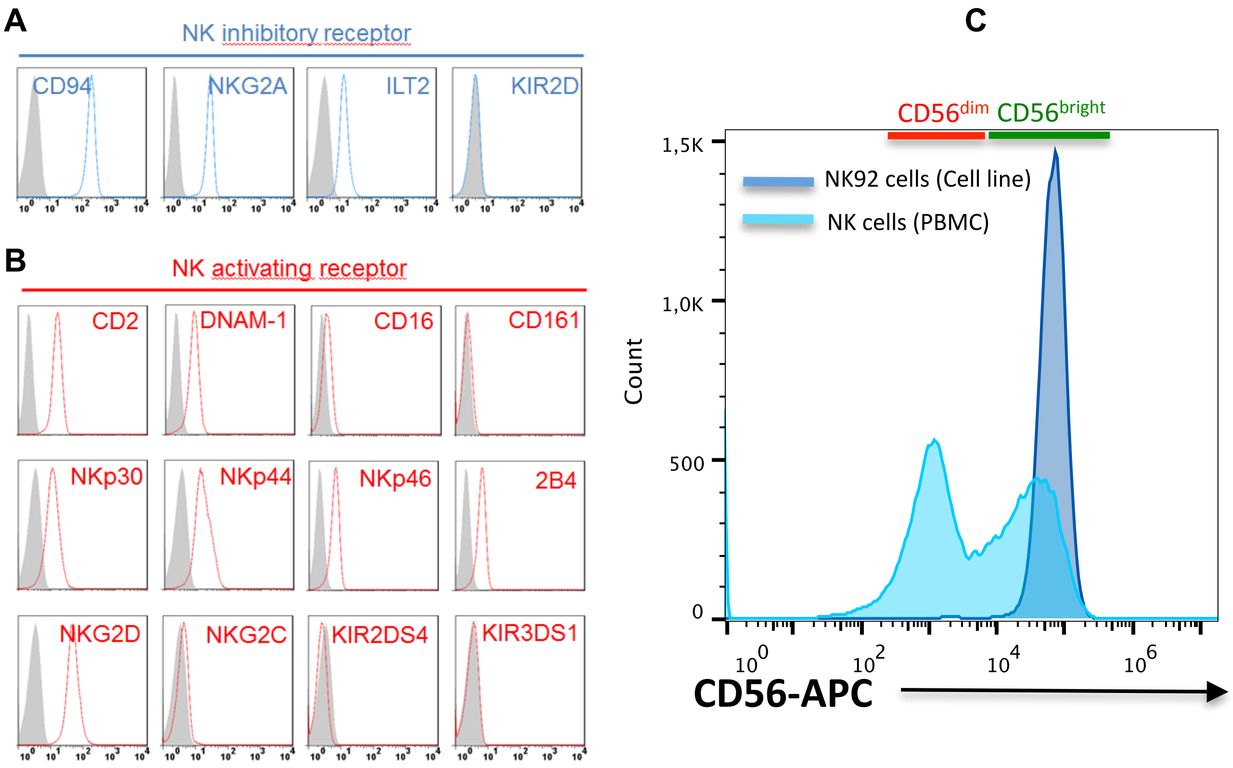

CD2	CD3	CD56	CD16	CD8	CD16	HLAC1	HLADR	DNAM1	CD94	NKG2A	NKG2C	NKG2D	NKp30	NKp44	NKp46	CD161	2B4	CD57	ILT2
+	-	+	-	-	-	+	-	$+_{low}$	+	+	-	+	$+_{low}$	$+_{low}$	$+_{low}$	-	$+_{low}$	-	+

$CD158_a$	$CD158_b$	p70	p50	KIR
-	-	-	-	-

Title Figure S3: Detailed phenotype of NK 92 cell line (Cytometry analysis)

Legend Supplementary Figure S3: Histograms illustrating NK inhibitory receptor (**A**) and activating receptor (**B**) expression on NK92 determined by flow cytometer (The profile of IgG isotype control is shown in grey filled histogram)**,** (**C**) Histograms illustrating $CD56^{pos}$ NK92 cells and $CD56^{pos}$ NK cells among PBMC (Cytometry analysis).

Supplementary Table S4

**GAPDH**	Forward primer	5′ CCCCTTCATTGACCTCAACTAC 3’
	Reverse primer	5′ GATGACAAGCTTCCCGTTCTC 3’
**INF-γ**	Forward primer	5’ CTAATTATTCGGTAACTGACTTGA 3’
	Reverse primer	5’ ACAGTTCAGCCATCACTTGGA 3’

Title table S4: PCR primers

Legend Supplementary table S4: GAPDH: Glyceraldehyde-3-phosphate dehydrogenase, **IFN-g:** Interferon gamma.

Supplementary Figure S5

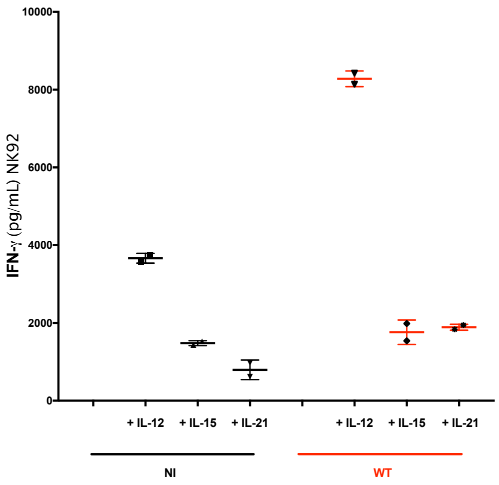

<u>Title Figure S5</u>: Influence of IL-12, IL-15 and IL-21 on IFN-g response after PA-WT infection

<u>Legend Supplementary Figure S5</u> IFN-g concentration was measured (ELISA) in supernatant of NK 92 cells after a 24-hour infection with or without IL-12 (5ng/ml) or IL-15 (50ng/mL, Miltenyi Biotec) or IL-21 (50ng/mL, Miltenyi Biotec) stimulation. WT: PA-WT 24-hour infection (Representative of 3 disctincts experiments)

Supplementary video S6

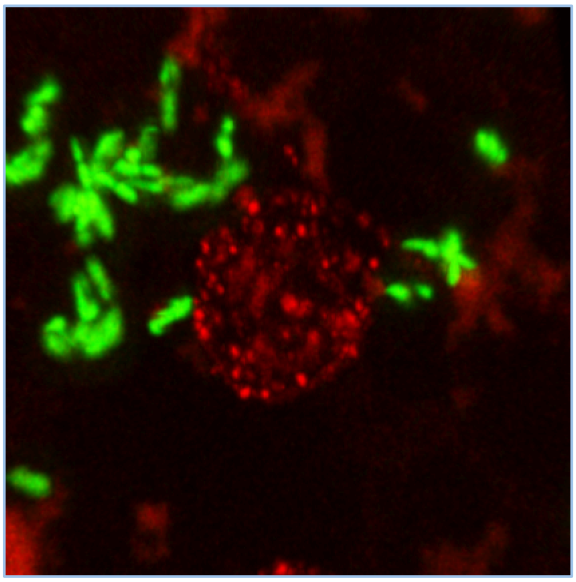

<u>Title video S6</u>: Live NK-PA-WT GFP interaction

<u>Video Legend Supplementary S6</u>: Picture of living confocal microscopy immediately after PA-WT-GFP challenge suggesting a direct bacteria-to-cell contact: NCR2 (Nkp44) are labelled in Red and PA-WT-GFP are labelled in green. **NCR**: Natural cytotoxic receptor. **PA-WT GFP**: PA wild-type strain expressing ExoS, T, Y, and the Green fluorescent protein. Projection of 9 steps of 2 µm on Z dimension. No other processing.

Supplementary Figure S7

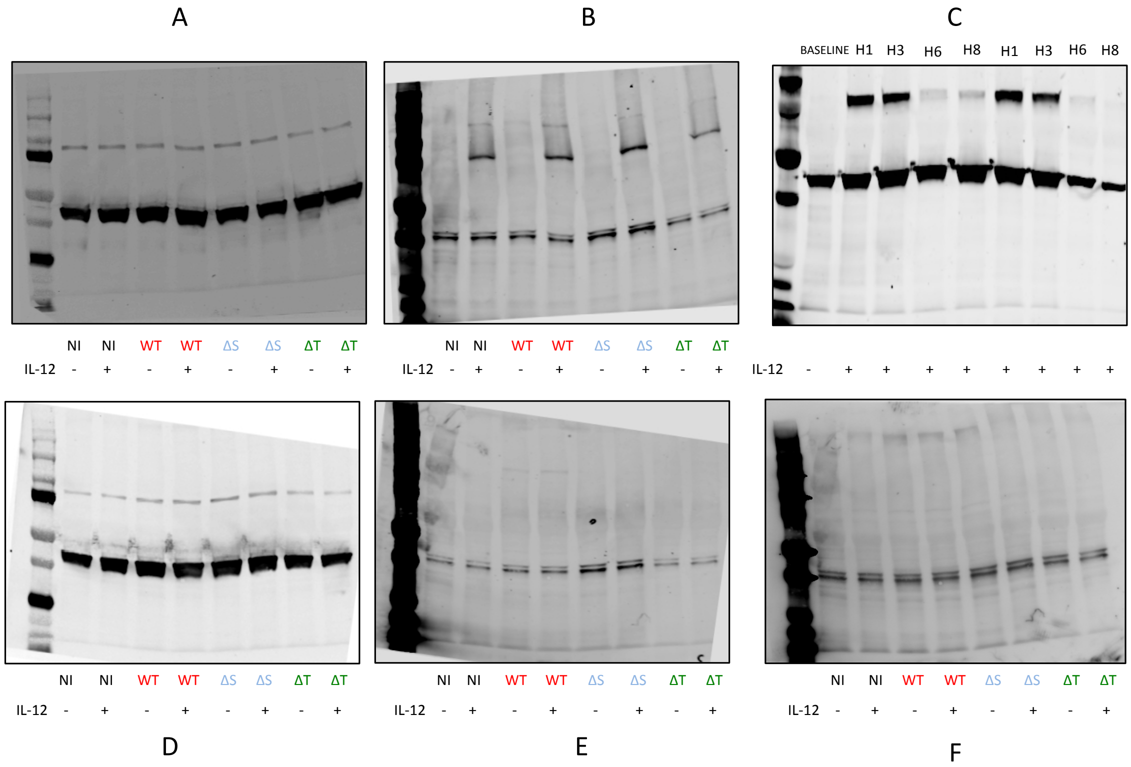

<u>Title: Figure S7:</u> Full-length Western Blot gels from Figure 2B,C and 3D.

<u>Legend Supplementary Figure S7:</u> Original gels were only cropped, flipped from right to left and rotated 180° when necessary to improve the clarity of the information. The (**A**) and (**B**) full-length gels stand for **Figure 2B**: (**A**) β-actin (45kDa) with 800nm and (**B**) P-stat 4 (81kDa) with 680 nm infrared radiation acquisition. The full-length gel (**C**) with P-stat 4 (81kDa) stands for **Figure 2C**. The (**D**),(**E**) and (**F**) full-length gels stand for **Figure 3D**: (**D**) β-actin (45 kDa) with 800 nm, (**E**) Phospho-p42/p44 (42,44 kDa) and (**F**) p42/p44 (42,44 kDa) with 680 nm infrared radiation acquisition.

Supplementary Figure S8

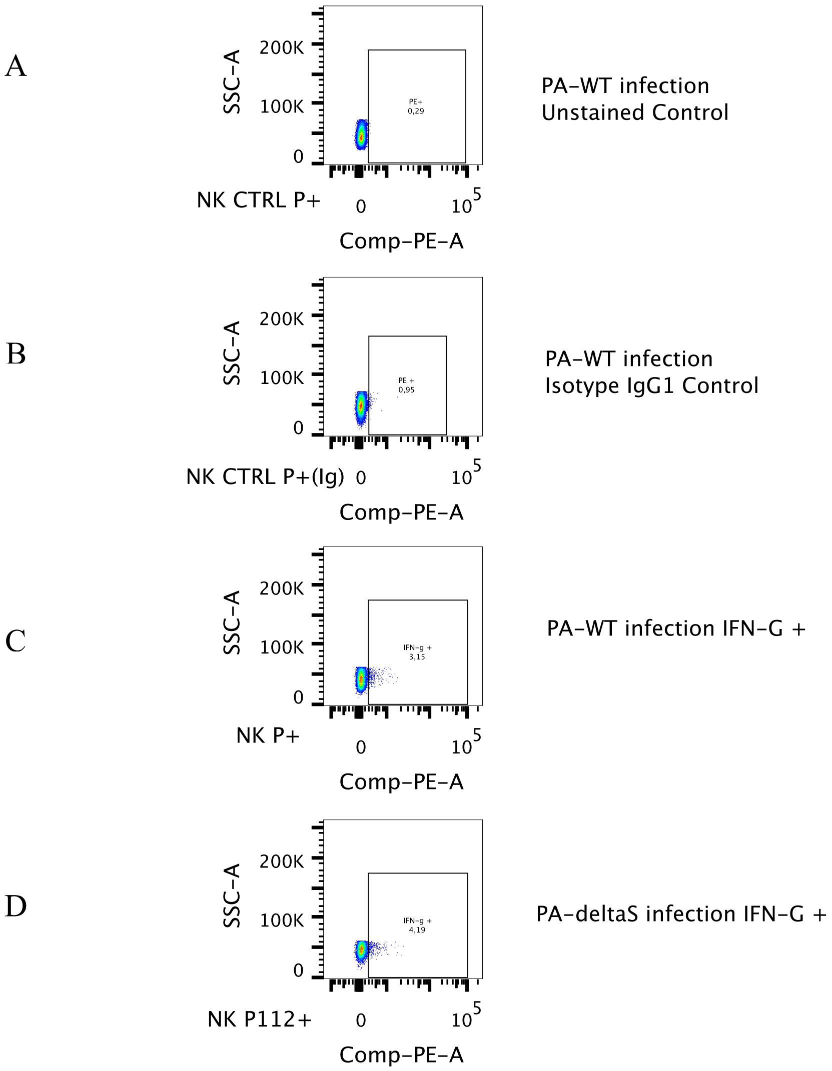

<u>Title: Figure S8:</u> IgG1 Isotype control profile for intracellular IFN-γ staining

<u>Legend Supplementary Figure S8:</u> Cytometry analysis of PBMC after 24-h PA or PA-ΔS infection. Representative density plot of Unstained control (A), intracellular staining with Isotype

control IgG1 (B), SSC/IFN-g$^+$-PE after PA infection (C) and SSC/IFN-g$^+$-PE after PA-ΔS infection (D) in sorted human NK cells.

Supplementary Figure S9

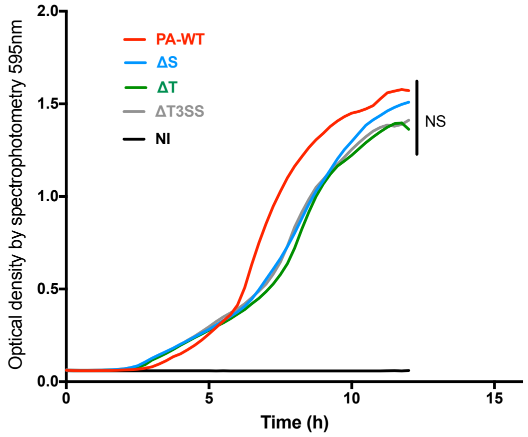

<u>Title Figure S9</u>: Growth curves of PA-WT and deleted strains

<u>Legend Supplementary Figure S9</u>: Evolution of the optical density (OD 595 nm) along 12 hours (37°C) of bacterial growth among PA-WT (WT) and deleted strains measured by spectrophotometry. Each strain was incubated in a well of a 96-well plate. The initial bacterial concentration was 1 x 10^6 UFC/mL

NS: Non-significant difference.

Supplementary Figure S10

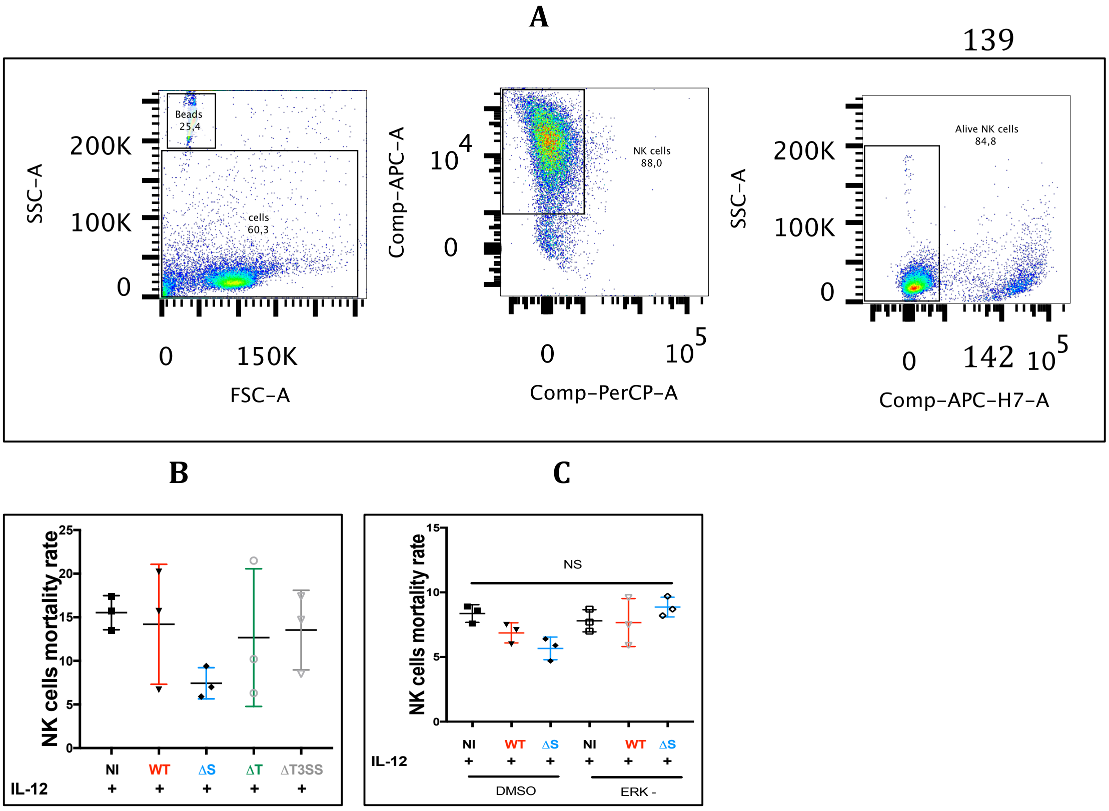

<u>Title Figure S10</u>: Mortality after PA infection among sorted human NK cells.

<u>Legend Supplementary Figure S10</u>: (A) Representative density plots after cytometry analysis illustrating APC H7-eFluor 780 positive among sorted human NK cell line ($CD56^{+}$APC/$CD3^{-}$ PerCP) after 2-hour PA-WT infection. (B) Representative histograms of mortality rate among sorted human NK cells after a 2-hour PA challenge with PA-WT or deleted strains after IL-12 stimulation. Data is presented as the median and interquartile range and is representative of 3 independent experiments (1 different healthy donor per experiment).

Supplemental Figure S11

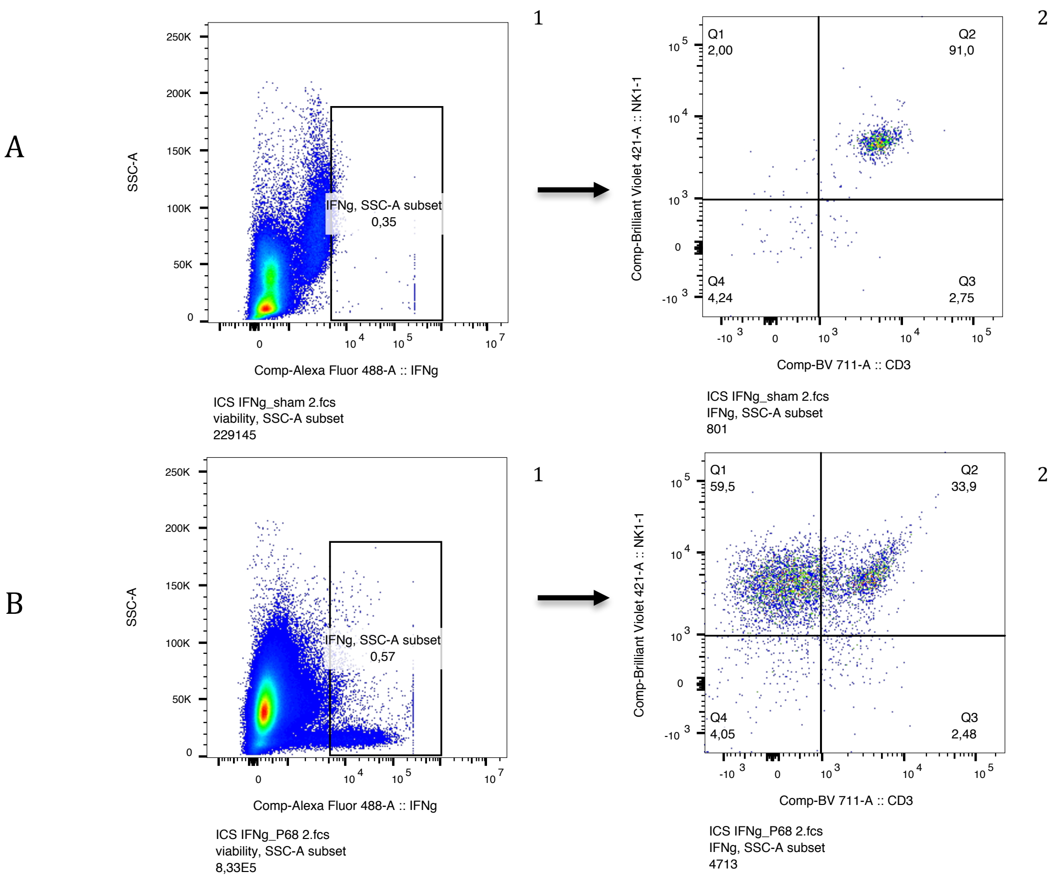

<u>Title Figure S11</u>: IFN-g source in lung after mouse-PA pneumonia

<u>Legend Supplementary Figure S11:</u> Cytometry analysis in Lungs after PA-WT pneumonia. Representative density plot of SSC/ IFN-g^{+}-FITC (1) and NK1.1/CD3 (2) in SHAM (A) or PA-WT Infected (B) mice. NK cells correspond to NK1.1^{+}-BV421/CD3^{-}APC cells and NKt cells correspond to NK1.1^{+}-BV421/CD3^{+}APC cells.
